# Targeting Infected Host Cell Heme Metabolism to Kill Malaria Parasites

**DOI:** 10.3390/ph19010167

**Published:** 2026-01-17

**Authors:** Faiza A. Siddiqui, Swamy R. Adapa, Xiaolian Li, Jun Miao, Liwang Cui, Rays H. Y. Jiang

**Affiliations:** 1 Department of Internal Medicine, Morsani College of Medicine, University of South Florida, Tampa, FL 33612, USA; faiza@usf.edu (F.A.S.); xli16@usf.edu (X.L.); jmiao1@usf.edu (J.M.); liwangcui@usf.edu (L.C.); 2 Global Health, College of Public Health, University of South Florida, Tampa, FL 33612, USA; swamyrakesh@usf.edu; 3 USF Genomics Program, University of South Florida, Tampa, FL 33612, USA

**Keywords:** malaria, erythrocyte, artemisinin, heme, porphyrin, protoporphyrin IX

## Abstract

**Background/Objectives**: Malaria remains a major global health burden, increasingly complicated by resistance to artemisinin-based therapies. Because artemisinin activation depends on heme and porphyrin chemistry, we sought to exploit host red blood cell (RBC) heme metabolism as a therapeutic vulnerability. This study aims to develop and evaluate a host-directed “bait-and-kill” strategy that selectively sensitizes malaria-infected RBCs to artemisinin. **Methods**: We integrated quantitative proteomics, erythropoiesis transcriptomic analyses, flow cytometry, and in vitro malaria culture assays to characterize heme metabolism in mature RBCs and Plasmodium falciparum-infected RBCs (iRBCs). The heme precursor 5-aminolevulinic acid (ALA) was used to induce porphyrin accumulation, and dihydroartemisinin (DHA) was applied as the killing agent. Drug synergy, porphyrin accumulation, reactive oxygen species (ROS) induction, and parasite survival were assessed, including ring-stage survival assays using artemisinin-resistant clinical isolates. **Results**: Mature RBCs retain a truncated heme biosynthesis pathway capable of accumulating porphyrin intermediates, while uninfected RBCs are impermeable to ALA. In contrast, iRBCs exhibit increased membrane permeability, allowing selective ALA uptake and porphyrin accumulation. ALA alone did not induce cytotoxicity or ROS, whereas DHA induced ROS and parasite killing. The ALA + DHA combination resulted in synergistic parasite elimination, including complete clearance of artemisinin-resistant *P. falciparum* isolates from the Greater Mekong Subregion, with no recrudescence observed over three weeks of culture. Evidence supports a predominant role for host-derived heme metabolites in mediating this synergy. **Conclusions**: The bait-and-kill strategy selectively exploits host RBC heme metabolism to restore and enhance artemisinin efficacy while sparing uninfected cells. Using clinically safe compounds, this host-directed approach provides a promising, resistance-bypassing framework for malaria treatment and combination drug development.

## 1. Introduction

Human red blood cells (RBCs) represent a terminally differentiated cellular entity characterized by the absence of an endomembrane system, nuclei, or mitochondria [1,2,3,4]. Because of their minimalist structure, RBCs possess unique metabolic properties that hold potential for the development of host-targeted therapies against malaria.

Heme metabolism in RBC plays a critical role in malaria [5], serving as the foundation for artemisinin activation [6] and hemozoin formation by the parasite. In human RBCs, the metabolic profile is characterized by a significant reliance on cytoplasmic pathways, particularly glycolysis, due to the absence of metabolic processes associated with cell division and mitochondrial-based energy or metabolite production [3,4]. Initially, young RBCs undertake heme biosynthesis through a two-part process involving both mitochondrial and cytoplasmic steps [7,8], intricately coordinated during erythroid development. However, upon maturation, only the cytoplasmic steps persist [3,4], presenting metabolic characteristics unique to mature RBCs.

Our recent studies have demonstrated the viability of a bait-and-kill strategy aimed at exploiting the metabolic vulnerabilities of cancer cells by leveraging their ‘truncated’ heme biosynthesis pathway [9,10,11]. Unlike normal cells, cancer cells possess this aberrant pathway, allowing for the accumulation of heme intermediates without complete conversion into the final product, heme. This strategy capitalizes on the clinical safety profile of ALA (Aminolaevulinic acid) [12,13], a heme precursor, which bypasses the initial mitochondrial step, thereby triggering the accumulation of heme intermediates, specifically porphyrins, within cancer cells. Subsequently, these accumulated porphyrins are targeted for elimination using compounds that induce redox stress [9]. Importantly, normal human cells, characterized by balanced heme pathways, remain unaffected due to their lack of porphyrin accumulation [9].

We propose that this strategy holds promise for malaria treatment due to three key factors: Firstly, mature RBCs inherently possess a truncated heme biosynthesis pathway [1,2,14]. Secondly, healthy/uninfected RBCs are impermeable to the heme precursor ALA, while infected RBCs demonstrate increased permeability [14,15,16,17,18], resulting in porphyrin accumulation. Thirdly, heme-mediated activation of artemisinin has been established as the major mechanism of action for its antimalarial activity [6]. Additionally, evidence from human ferrochelatase (last step of heme biosynthesis)-deficient erythropoietic protoporphyria patients indicates resistance to malarial parasite growth, suggesting that natural human genetic variation in aberrant heme metabolism can confer malaria resistance [19].

With the emergence of artemisinin resistance [20,21,22], there is an urgent imperative to explore novel and safe antimalarial drugs. In this study, we use the latest quantitative proteomics and erythropoiesis data to elucidate the unique heme pathway of RBCs in their terminally differentiated state. Contrary to previous antimalarial work involving light as PDT (Photodynamic Therapy) therapy [14], or using human proteins targeting circulating heme [23], our approach is entirely based on clinically safe drugs. In agreement with and building upon previously published results by Sigala et al. [14], we found RBC heme metabolism is intrinsically distinct compared to other human cells that can be used for host metabolic targeting. We demonstrate the synergy between ALA and artemisinin, which can be utilized to target clinically obtained artemisinin-resistant parasites.

## 2. Results

### 2.1. Result 1: RBC Possesses a Unique Truncated Heme Biosynthesis Pathway

**Figure 1:**
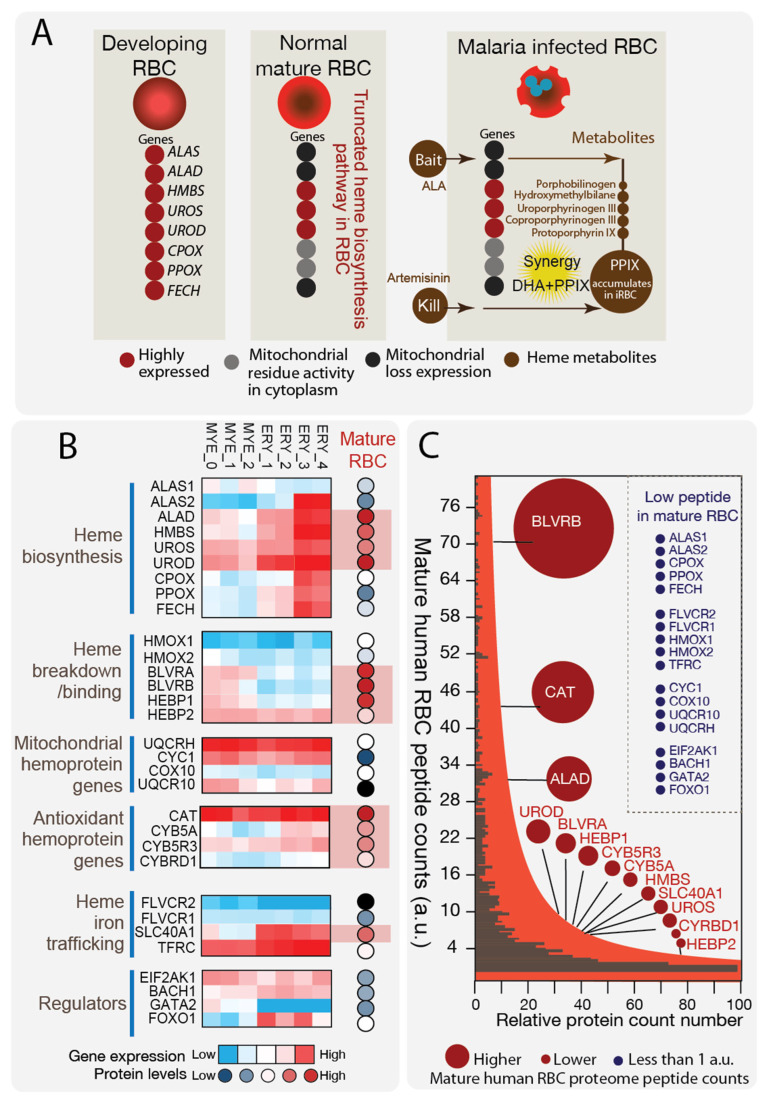
Erythropoiesis Gene Expression and Mature RBC Proteome Analysis. (**A**). Schematic representation illustrating the heme biosynthesis pathway in developing versus terminally differentiated stages. Upon erythroid maturation, the loss of cell organelles including mitochondria results in the diminished presence of mitochondrial heme biosynthesis enzymes. Evidence of residual functions of the last steps has been found in in-depth analysis of the proteome of mature human RBCs, as well as previously demonstrated (Sigala et al.). Right panel: Malaria-infected RBCs can uptake the heme precursor ALA and accumulate porphyrins. (**B**). Human erythropoiesis gene expression analysis reveals upregulation of the entire heme biosynthesis pathway into the last stages before maturation. The erythropoiesis stages are: common myeloid progenitor (MYE_0), megakaryocyte/erythroid progenitor (MYE_1 and MYE_2), and Erythroid cells (ERY1-4). Mitochondrial heme protein genes, such as *UQCRH* and *CYC1*, are robustly expressed. For heme synthesis related trafficking, there is concurrent upregulation of iron import (*TFRC*) and export (*SLC40A1*) genes. Heme breakdown is downregulated. On the right panel, by contrast, the fully mature RBC proteome shows abundant cytoplasmic heme biosynthesis enzymes, including ALAD, HMBS, UROS, and UROD, while mitochondrial enzymes are depleted. Iron export (SLC40A1) and heme degradation proteins (BLVRB, BLVRA) are present at high levels in mature RBCs, whereas iron import and mitochondrial located heme biosynthesis proteins (ALAS1, ALAS2, CPOX, PPOX, and FECH) are greatly reduced. (**C**). Mid-step heme biosynthesis genes and heme degradation genes are among the highest-level proteins in mature RBCs, while first or last heme biosynthesis genes are present at low levels or are undetectable, indicating an intrinsic truncated pathway present in normal human mature RBCs. RBC hemoproteins responsible for antioxidant responses, such as catalase (CAT), cytochrome b5 reductase A (CYB5A), and cytochrome b5 reductase D (CYRBD1), are prominently expressed in the mature proteome, indicating the importance in detoxifying radical species. Heme degradation and iron export proteins are present, suggesting breakdown during the RBC lifespan during the halting of heme biosynthesis. Mitochondrial respiratory hemoproteins and heme regulators during development are present at very low levels, confirming extrusion of mitochondria and cessation of key erythroid developmental processes.

Human RBCs are terminally differentiated cells characterized by distinct proteomic and metabolic profiles [3,4]. This distinctiveness in heme biosynthesis enzymes and the resulting substrate production offers a promising approach for targeting malaria parasites by disrupting the unique host heme metabolism (Figure 1A). Porphyrin production in cancers, a consequence of this unique pathway [9], exhibits redox activity [24] and can be exploited to selectively eliminate pathological cells. Our rationale is that we can employ a similar approach to kill malaria-infected RBCs due to the similarity in the ‘truncated’ heme biosynthesis pathways present in both cancers and RBCs.

First, we conducted terminal erythropoiesis analysis, based on previously published human hematopoiesis data [25] and quantitative proteomic analysis of mature RBCs [2], to elucidate the heme metabolic processes in the mature cell stage. Our aim was to better understand the metabolic vulnerability of host cells during *P. falciparum* infection. Utilizing human hematopoiesis gene expression data, we examined the expression of genes involved in heme metabolic processes across various erythroid progenitor stages (Figure 1B) (Supplementary Materials Table S1). Our analysis encompassed the different temporal steps of erythropoiesis, including common myeloid progenitor (MYE_0), megakaryocyte/erythroid progenitor (MYE_1 and MYE_2), and erythroid cells (ERY1-4). We investigated various key aspects of heme metabolism in RBCs, including heme biosynthesis pathway genes, heme degradation and binding processes, mitochondrial hemoprotein genes, heme/iron trafficking genes. We also examined erythropoiesis principal regulator genes, such as *EIF2K1* (heme regulated global translation initiation kinase) [26,27], *BACH1* (heme regulated transcription activator) [28], *GATA2* (early erythropoiesis regulator) [29,30] and *FOXO1* (late erythropoiesis regulator) [31,32]. Consistent with established findings [25], we observed distinct gene expression patterns in the heme biosynthesis pathway during erythrocyte maturation. We noted a progressive upregulation of the heme biosynthesis pathway, reaching its peak in the later stages of maturation with high levels of expression of all eight steps of genes. Particularly noteworthy were the robust expression levels of key mitochondrial hemoprotein encoding genes, such as *UQCRH* and *CYC1,* which play crucial roles in powering cellular respiration, during erythroid differentiation. This highlights the significance of mitochondrial metabolism and electron transport chain function in erythroid development and differentiation. Furthermore, our analysis revealed a coordinated upregulation of iron import gene *TFRC*, indicative of fueling hemoglobin synthesis, and iron export gene SLC40A1, to maintain heme homeostasis. This was accompanied by a downregulation of heme breakdown pathways, *BLVRA* and *BLVRB*, indicative of active heme production and its incorporation into cellular components during erythroid differentiation.

Subsequent to RBC maturation, the expulsion of DNA regulatory machinery and elimination of subcellular organelles predominates. Examination of recent quantitative RBC proteomic data [1,2] delineates the transition from late differentiated erythroid to fully mature RBCs (Figure 1C) (Supplementary Materials Table S2). Mature RBCs exhibit minimal expression levels of mitochondrial hemoproteins (UQCRH and CYC1) and master regulators of heme metabolism (EIF2K1 and BACH1) during differentiation, indicating a cessation of both nuclear and mitochondrial metabolism and heme-regulated developmental processes in mature proteome. Notably, significant reductions are observed in both the initial (ALAS2) and final steps (CPOX, PPOX, and FECH) of heme biosynthesis occurring in mitochondria, with levels low or undetectable per cell. In contrast, mid-step cytosolic enzymes (ALAD, HMBS, UROS, and UROD) persist at considerable levels (7000 to 800,000 copies protein per cell) in two independently generated proteome datasets, suggesting that only the mid-steps of heme biosynthesis enzymes constitute part of the canonical proteome within mature RBCs. This indicates an intrinsically truncated heme biosynthesis pathway akin to our findings in cancer cells, predisposing the pathway to the accumulation of intermediates. Additionally, the presence of low levels of iron import proteins (TFRC) and high levels of iron export (SLC40A1) and heme degradation proteins (BLVRA and BLVRB) further supports the absence of heme biosynthesis but the maintenance of heme breakdown during the mature RBC lifespan. Furthermore, RBC hemoproteins responsible for antioxidant responses [33,34,35], such as catalase (CAT), cytochrome b5 reductase A (CYB5A), and cytochrome b5 reductase D (CYRBD1), are prominently expressed in the mature proteome, indicating their crucial role in detoxifying radical species and maintaining RBC integrity and function against the onslaught of redox stress during RBC circulation.

### 2.2. Result 2: iRBC in Dihydroartemisinin (DHA) Resistance Stage Are Remodeled for Host Cell Permeability Change

The emergence of artemisinin resistance in malaria parasites has raised significant concerns in recent years. Notably, key genes associated with artemisinin resistance, such as *Kelch 13* [20,36], ubiquitin hydrolase (*UBP1*) [37], AP2 adaptor complex μ-subunit (*AP2-MU*) [38,39], Kelch13 interaction candidate (*KIC5*) [40], and Kelch13 interaction candidate (*KIC7*) [41], exhibit enrichment of expression in the ring stage of infection [20,36], suggesting their potential involvement in resistance mechanisms during the early stages of parasite colonization. To combat drug resistance effectively, targeting infected host cells during this critical stage becomes imperative. We hypothesize that infected RBCs provide an optimal environment for inducing porphyrin accumulation due to their increased permeability, the precursor of heme intermediates, facilitated by parasite-induced changes in host cell permeability [42,43].

**Figure 2:**
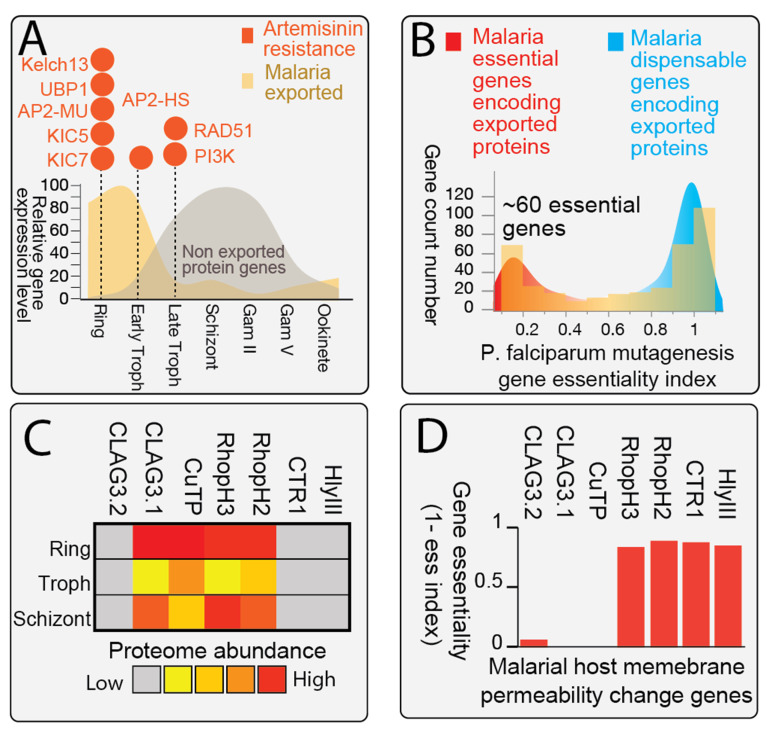
Gene Expression and Proteomic Evidence of *P. falciparum* Parasite Inducing Host Cell Permeability Changes During Erythrocytic Stage. (**A**). Predicted exported proteins (>400) by *P. falciparum* during the blood stage growth exhibit a peak gene expression pattern at the ring and early trophozoite stages. In contrast, non-exported protein-encoding genes show a peak expression pattern in the late trophozoite and schizont stages. Several previously reported key players of artemisinin resistance-related genes, such as *K13*, *UBP1*, *AP2-MU*, *KIC5*, and *KIC7*, are enriched in the ring stage of infection. (**B**). *P. falciparum* exported protein-encoding genes display a bimodal forward genetic gene essentiality pattern, as evidenced by transposon mutagenesis phenotype survival data. While the majority of exported protein genes are classified as non-essential during in vitro blood stage culture, a subset of 60 exported protein genes are phenotyped as essential for blood stage parasite survival, indicating their importance in the export and host remodeling process. (**C**). A set of previously published parasite-encoded genes that modulate host RBC permeability are upregulated in the ring stage. Whole proteome protein abundance data reveal that *CLAG3.1*, *CuTP*, *RhopH2*, and *RhopH3* proteins are highly expressed in the ring stage. (**D**). A subset of parasite-host permeability-changing genes, including *RhopH2*, *RhopH3*, *CTR1*, and *HlyIII*, demonstrate forward genetic growth essentiality during random transposon mutagenesis under standard in vitro blood stage culture conditions, indicating the significance of host cell remodeling for parasite survival within RBCs.

We investigated the gene expression and proteomic evidence of *P. falciparum* parasites inducing changes in host cell permeability during the erythrocytic stage, with a specific focus on alterations in infected RBCs (iRBCs) associated with DHA resistance (Figure 2A) (Supplementary Materials Table S3). Initially, we obtained a predicted set of 386 parasite-exported proteins from PlasmoDB v68, which revealed a distinct gene expression pattern of protein export during blood stage growth. Notably, we observed a peak in gene expression occurring at the ring and early trophozoite stages in the blood stage expression dataset [44]. Intriguingly, this timeframe coincided with the expression of genes associated with artemisinin resistance. This synchrony suggests a potential opportunity to leverage enhanced host permeability as a strategy for targeting drug resistance.

To evaluate the essentiality of the export process, we examined *P. falciparum* exported protein-encoding genes in the context of saturation mutagenesis through transposon tagging (Figure 2B). The mutagenesis index, reflecting the essentiality of in vitro blood stage parasite survival, indicated that lower values correspond to greater essentiality [45]. Our analysis unveiled a bimodal forward genetic gene essentiality pattern, with approximately 60 exported protein genes identified as essential for blood stage parasite survival. This suggests their critical role in host remodeling and export mechanisms. The identification of these essential genes further supports the rationale for targeting this process as a potential antimalarial strategy.

Moreover, we investigated parasite-encoded genes implicated in modulating host RBC permeability, such as *CLAG3.1* [46], *CuTP* [47], *RhopH2* [48], and *RhopH3* [49], alongside *CTR1* [50] and *HlyIII* [51], to understand their expression patterns and genetic essentiality. Our analysis revealed that during the ring stage of infection, four key proteins, CLAG3.1, CuTP, RhopH2, and RhopH3, are highly expressed at the protein level (Figure 2C). Additionally, a subset of these genes, including *RhopH2*, *RhopH3*, *CTR1*, and *HlyIII*, demonstrated forward genetic growth essentiality, indicating the critical role of host cell remodeling for parasite survival within RBCs (Figure 2D). Taken together, these findings highlight the potential to leverage host permeability changes induced by infection as a strategy for targeting the host heme metabolism.

### 2.3. Result 3: Normal Blood Cells Do Not Accumulate Porphyrin

**Figure 3:**
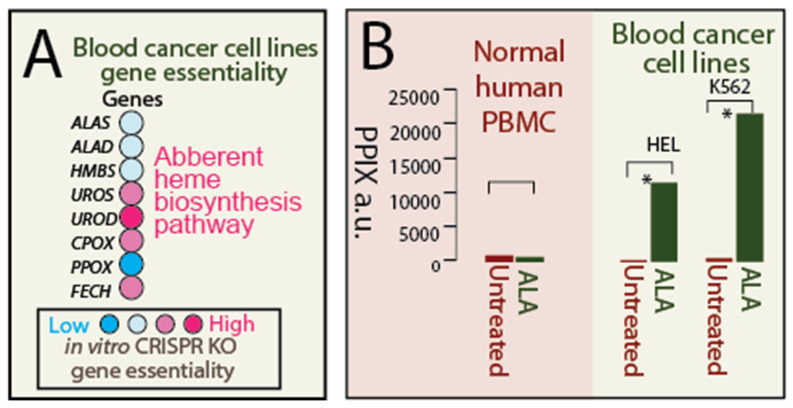
Accumulation of Protoporphyrin IX (PPIX) Is Absent in Normal Human Peripheral Blood Mononuclear Cells (PBMCs). (**A**). Blood cancer cell lines exhibit an aberrant heme biosynthesis pathway, similar to the heme biosynthesis pathway found in mature human RBCs. in vitro CRISPR knockout (KO) gene essentiality analysis reveals that mid-step heme biosynthesis genes, particularly UROD, are crucial for the survival of blood cancer cells. In contrast, the initial and later steps of the pathway show lower essentiality, indicating an imbalanced heme biosynthesis process that leads to the accumulation of heme intermediates such as Protoporphyrin IX (PPIX). This imbalance mirrors the ‘truncated’ heme biosynthesis observed in normal mature human RBCs. (**B**). Normal human peripheral blood mononuclear cells (PBMCs) do not accumulate PPIX, with or without ALA, indicating strict heme biosynthesis control and absence of intermediate buildup. * indicates significant differences in over 50,000 cells counting with *p* value < 0.0001). ALA is either efficiently converted to the final heme product or degraded in normal human PBMCs. As a control, the blood cancer cell lines HEL and K562 robustly accumulate porphyrins to thousands-fold higher levels after adding ALA.

Our recent study targeting the aberrant heme biosynthesis pathway in cancer cells has prompted us to investigate a similar pathway in normal human mature RBCs. We observed that blood cancers share a similar ‘truncated’ heme biosynthesis pathway with mature human RBCs. To validate this observation, we analyzed in vitro CRISPR knockout essentiality data from leukemic cell lines, leveraging the Cancer Dependency Map (DepMap) dataset (23Q4). This dataset provides information on genetic dependence inferred from cell survival upon specific gene knockout, allowing us to assess the importance of heme biosynthesis pathway genes in blood cancer cells (Figure 3A). We found that mid-step heme biosynthesis genes, particularly UROD, were crucial for blood cancer cell survival, highlighting their role in sustaining the dysregulated heme biosynthesis process, which parallels the abundance of mid-step enzymes in human mature RBCs. In contrast, the genes involved in the initial and final steps of the pathway exhibited lower essentiality, which parallels the limited presence of these enzymes in human mature RBCs. These analyses highlight heme metabolic vulnerabilities in blood cancer cells that resemble those observed in human mature RBCs, providing a rationale for targeted therapeutic interventions.

Our anti-cancer strategy relies on the premise that normal cells do not accumulate porphyrins, even under ALA induction, providing a therapeutic window for selective targeting of cancer cells. Similarly, to target malaria-infected cells, we conducted further experimentation to demonstrate that normal human blood cells cannot be induced to accumulate porphyrins.

We performed ALA induction experiments on normal human PBMCs (Peripheral Blood Mononuclear Cells) to elucidate heme biosynthesis dynamics (Figure 3B). Normal PBMCs did not show any accumulation of PPIX (Protoporphyrin IX), regardless of ALA presence, indicating stringent control of heme biosynthesis and the absence of intermediate buildup. As a control, blood cancer cell lines robustly accumulated porphyrins upon ALA addition, reflecting the aberrant heme intermediate accumulation in these cells. These findings highlight the specificity and safety of our targeting strategy, as the diverse normal blood cell populations represented by PBMC do not accumulate porphyrins.

### 2.4. Result 4: iRBCs Specifically Accumulate Porphyrin

**Figure 4:**
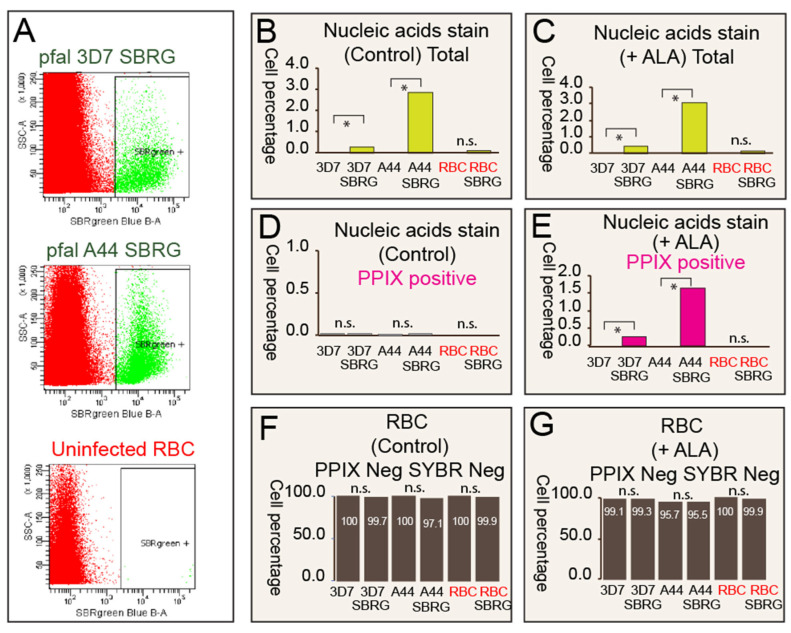
P. falciparum-Infected RBCs Accumulate Porphyrins upon Adding ALA. Two parasite strains, *P. falciparum* 3D7 and A44 (A44 refers to the clinical strain F09A44), were tested for PPIX accumulation during RBC infection in vitro. (**A**): Flow cytometry scatterplot separating infected RBCs from uninfected RBCs using SYBR Green (SRBG) nucleic acid signal. The *Y*-axis represents flow cytometry Side Scatter (SSC), and the *X*-axis represents SYBR levels. (**B**): SRBG staining reveals varying levels of parasitemia with 3D7 and A44 in vitro infections. (**C**): Upon adding ALA, similar percentages of SRBG-positive (infected) RBCs were found. (**D**): Without adding ALA, no PPIX-positive cell populations were detected in infected RBCs or control normal RBCs. (**E**): Both 3D7 and A44 infected cells accumulated PPIX upon ALA addition. (**F**): Without ALA, most RBCs are negative for PPIX and SYBR, with a slight reduction in PPIX and SYBR negativity observed in infected RBCs due to parasite nucleic acid content. (**G**): Upon ALA addition, most RBCs are negative for PPIX and SYBR. PPIX signal was visualized using excitation at 405 nm and emission in the red channel at 633 nm. SYBR Green dye was used to label nucleic acids, with excitation and emission at 498/522 nm. n.s. indicates not significant. * indicates significant differences in over 50,000 cells counting with *p* value < 0.0001).

To demonstrate the accumulation of PPIX within infected red blood cells (iRBCs) during malaria infection, we used two strains of malaria parasites to infect human RBCs (Figure 4A–C)). We specifically tracked the parasites in the trophozoite stage, characterized by increased parasite biomass and clearer marker visualization. Two strains of the malaria parasite, *P. falciparum* 3D7 and F09A44, were tested for PPIX accumulation. 3D7 is a well-established laboratory-adapted strain, known for its consistent growth characteristics and documented susceptibility to antimalarial drugs. F09A44 is a clinical isolate obtained from the China-Myanmar area in 2009 and meets the WHO criteria for artemisinin resistance. The designation “F09” indicates the year of isolation. F09A44 carries the C469Y mutation in the *K13* gene, which is associated with artemisinin resistance. This mutation resulted in approximately 10% RSA (Ring Survival Assay) values in our initial experiments after the strain was adapted to the laboratory. The isolate was also used in a previous publication to study *K13* polymorphism [52]. PPIX fluorescence was detected using excitation at 405 nm and emission in the red channel at 633 nm. Additionally, SYBR Green dye, with excitation and emission at 498/522 nm, labeled nucleic acids of the parasites (Figure 4A–C). Normal RBCs (>99.9%) did not exhibit PPIX accumulation regardless of ALA presence. For 3D7-infected RBCs with lower parasitemia, PPIX accumulation was absent without ALA but increased upon ALA addition (Figure 4D,E). Similarly, F09A44-infected RBCs with higher parasitemia showed no PPIX accumulation without ALA, but levels rose after ALA addition (Figure 4D,E). The difference between Figure 4C (total infected cells) and Figure 4E (infected cells positive for PPIX) arises because not all infected cells meet the criteria for high levels of PPIX positivity. Despite this, the data demonstrate that PPIX positivity closely tracks infection levels. Importantly, the majority of RBCs, both in the total normal population and uninfected subpopulations, did not accumulate significant amounts of PPIX (Figure 4F,G). Unlike all nucleated healthy human cell types we tested [9,10,11], a small increase in some uninfected RBCs co-cultured with infected RBCs was observed, possibly due to parasite-released micro-vesicles [53]. However, no hemolysis was observed in uninfected cells, consistent with decades of ALA human clinical safe use without reported hematological effects. Taken together, these findings suggest that active malaria infection facilitates ALA entry and subsequent heme precursor conversion, bypassing the initial mitochondrial step and leading to porphyrin accumulation in infected RBCs.

### 2.5. Result 5: DHA and ALA Synergy Study

To explore the potential synergy between DHA and ALA in targeting porphyrin accumulation, we conducted a series of experiments using cell viability assays. As RBCs are terminally differentiated and unsuitable for quantitative drug synergy assays, we utilized the HC-04 liver cancer cell line [54,55,56] to demonstrate the synergy.

We conduct experiments to quantify the cytotoxic effects of ALA and DHA, both individually and in combination, across a broad range of drug concentrations (Supplementary Materials Figure S1). We assessed a wide spectrum of drug concentrations, ranging from nanomolar (nM) to 133 micromolar (μM) concentrations of DHA and from nanomolar (nM) to millimolar (mM) concentrations of ALA. This drug–drug interaction study is performed in experimental triplicates (Supplementary Materials Table S4). Our findings revealed that while treatment with either ALA or DHA alone demonstrated minimal toxicity, their combined administration exhibited a robust synergy in killing cancer cells. Remarkably, this synergistic effect was evident even at low concentrations of DHA in the nanomolar (nM) range. This observation suggests that ALA sensitizes DHA, likely through the accumulation of PPIX, thereby enhancing its efficacy in targeting and eliminating cancer cells. Notably, nanomolar concentrations of DHA proved sufficient for inducing cell death in this cell line assay, following sensitization by ALA, indicating the potential of this strategy to overcome malaria artemisinin resistance.

The bait-and-kill strategy exhibited a favorable safety profile with no evidence of off-target cytotoxicity. Previous evaluation of the ALA + DHA combination in normal human fibroblasts showed no detectable toxicity at concentrations comparable to those used in this study [9]. In addition, independent in vivo and organoid studies have demonstrated that systemic ALA combined with artemisinin derivatives does not induce toxicity in healthy tissues, while retaining selective activity in pathological contexts [57]. These findings support the non-toxic and host-directed nature of the bait-and-kill approach.

To further investigate the mechanistic basis of the bait-and-kill strategy, we performed reactive oxygen species (ROS) quantification assays to evaluate redox stress in treated cells. Across multiple biological replicates (n > 3), we observed an increase in ROS levels following DHA treatment, with similarly elevated ROS levels in the ALA + DHA group (Supplementary Materials Figure S2). These results confirm that DHA induces strong oxidative stress. However, due to the high baseline ROS from DHA alone, additional refined assays will be required to clearly delineate the specific contribution of ALA in enhancing ROS formation.

### 2.6. Result 6: DHA and ALA Kill DHA-Resistant Parasites

Based on our investigation into RBC heme metabolism and DHA sensitization, we propose a Bait-and-Kill Strategy to combat artemisinin-resistant malaria, utilizing ALA as the “bait” and artemisinin as the ‘kill’ agent. Given the global impact of malaria, the success of this strategy hinges on the safety profile of ALA. Notably, ALA has been employed as an FDA-approved cancer imaging agent since 2007, with extensive use in Europe preceding its adoption in the United States. Over 58,000 individuals worldwide have used ALA with no discernible adverse effects. ALA is routinely utilized in mouse models for both imaging and treatment, with a good safety profile [12]. Studies in Japan have examined its long-term use for up to three months [13] and large dose administration of up to 2250 mg/day without reported toxicity [58]. Presently, ALA is marketed as a nutritional supplement in Japan (SB Pharma). Human pharmacodynamic studies [59,60] have directly monitored ALA metabolism, revealing no toxic effects. PPIX, metabolized from exogenous ALA, can persist in human sera for several hours before complete elimination, typically occurring within 35–48 h. Detailed information on ALA’s clinical use, safety profile, and human pharmacokinetics is provided in Supplementary Table S5. Collectively, ALA’s widespread use across diverse populations suggests its safety for use as a public health intervention against infectious diseases.

**Figure 5:**
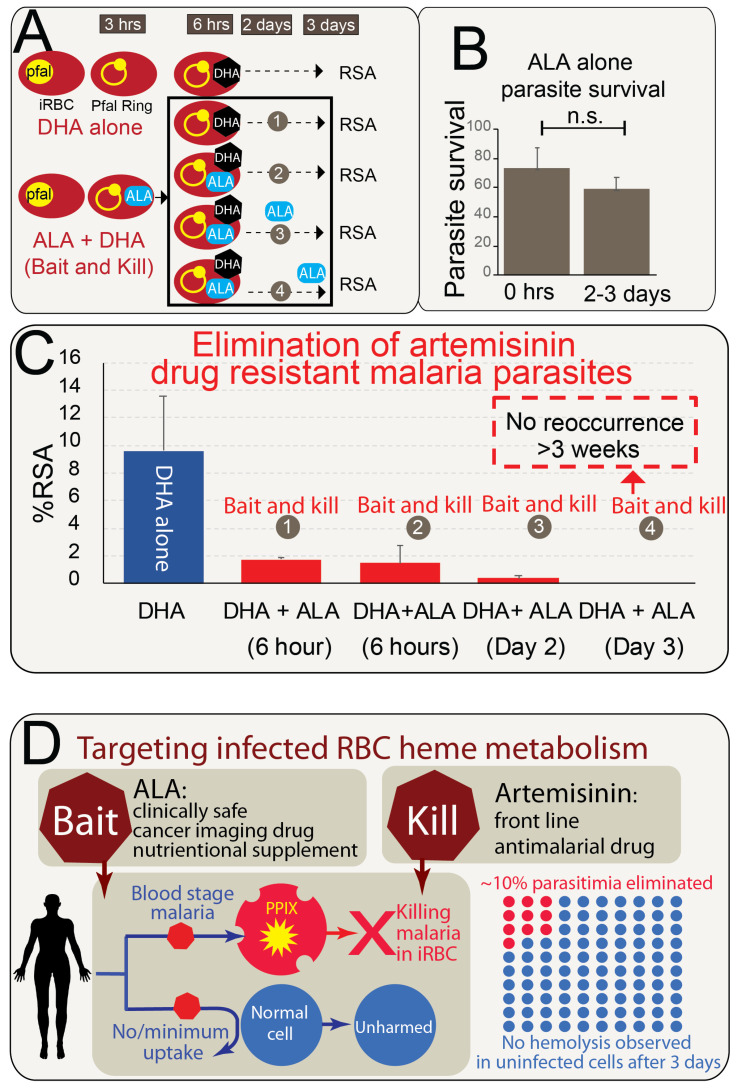
Killing Artemisinin-Resistant Malaria with Bait-and-Kill Strategy. (**A**). Schematic representation of the bait-and-kill assay, illustrating the experimental setup and methodology. (**B**). The effect of ALA alone on parasite elimination is not significant (n.s.) under the conditions tested. Synchronized ring-stage parasites were treated with 1 mM ALA alone, either as a single dose or replenished daily for days, mimicking the combination treatment protocol. Parasitemia was assessed by microscopy after >48 h of culture. Each condition was tested in four biological replicates (n = 4). No significant reduction in parasitemia was observed in ALA-only treated wells compared to untreated controls. Error bars represent standard error of the mean (SEM). (**C**). Artemisinin-resistant clinical isolates were effectively targeted using the bait-and-kill strategy, which synergizes ALA and DHA. The experiment utilized the clinical artemisinin-resistant parasite F09A44. Results from the Ring Survival Assay (RSA) showed that DHA alone led to approximately 10% parasite survival, while the combination of ALA + DHA reduced parasite survival to around 1% by day 3. Long-term culture confirmed complete parasite elimination, with no parasite recrudescence observed after 3 weeks of continuous monitoring. This outcome is visually indicated by a dashed red-line box, denoting the absence of parasite outgrowth in all replicates during the extended culture period over weeks. (**D**). Schematic representation of the proposed bait-and-kill strategy for targeting artemisinin-resistant parasites. Uninfected red blood cells (RBCs) do not uptake ALA and thus do not produce Protoporphyrin IX (PPIX), depicted in red. In contrast, infected RBCs uptake ALA, leading to PPIX production. This approach selectively targets malaria-infected red blood cells, as ALA is taken up by these cells and most cannot enter normal RBCs. The metabolism of human RBCs results in the production of porphyrins (PPIX) only in infected cells. Artemisinin synergizes with porphyrin to effectively kill malaria parasites. Bottom Right: In the experiments in panel A, uninfected RBCs are not observed to be affected after 3 days of culture with a high concentration of 1 mM ALA.

To evaluate the Bait-and-Kill Strategy against artemisinin-resistant clinical isolates, we used the stringent artemisinin resistance assay RSA (Figure 5A). We utilized the clinical isolate F09A44 (a Southeast Asian clinical isolate) carrying the K13 (C469Y) mutation associated with drug resistance. Characterized as artemisinin-resistant, the Ring Survival Assay (RSA) revealed that 700 nM DHA alone resulted in approximately 10% parasite survival. To demonstrate that ALA alone does not exert significant antimalarial activity, we show in Figure 5B that treatment with ALA (1 mM) does not lead to a significant reduction in parasitemia. Even after extended culture for more than two days (n = 4 replicates), no appreciable decrease in parasite burden was observed.

Subsequently, we applied a combination drug therapy comprising ALA and DHA (Figure 5C). Initially, 1 mM ALA was administered to facilitate PPIX accumulation, followed by the addition of 700 nM DHA for parasite eradication. ALA was replenished at only 6 h (wash out time of DHA), or two days or all three days until 72 h, the readout time for RSA. Our results demonstrated that the combination of ALA and DHA when ALA was replenished everyday resulted in complete parasite elimination by day 3, with no recurrence observed after 3 weeks of continued culture. The experiment was conducted in biological replicates (n = 3), providing evidence that this strategy effectively eradicates resistant parasites without recrudescence from parasite dormancy.

In summary, our proposed Bait-and-Kill Strategy targets artemisinin-resistant parasites (Figure 5D. We show that uninfected red blood cells (RBCs) do not uptake ALA and therefore do not produce PPIX, remaining unharmed by the drug combination. In contrast, infected RBCs uptake ALA, leading to PPIX production, sensitizing DHA to kill the parasite. This approach selectively targets malaria-infected red blood cells, as ALA is exclusively taken up by these cells and cannot enter normal RBCs.

## 3. Discussion

Amidst the persistent threat of malaria and the rise of artemisinin resistance, urgent and innovative solutions are imperative. Drawing from our recent discoveries concerning cancer heme metabolic vulnerability, we have introduced a novel therapeutic strategy termed the “bait-and-kill” approach. This strategy targets analogous metabolic susceptibilities found in both malaria-infected red blood cells (iRBCs) and cancer cells. By exploiting the truncated heme biosynthesis pathway in mature RBCs and the heightened permeability of infected cells to the heme precursor ALA, we have effectively demonstrated the potent synergy between ALA and artemisinin in eradicating artemisinin-resistant Southeast Asian clinical isolates. Significantly, our strategy provides a targeted intervention that selectively eliminates infected RBCs while preserving uninfected cells, thereby minimizing collateral damage. These findings offer promise for the development of large-scale public health interventions leveraging clinically safe drugs.

In our study, we show the importance of considering human erythroid heme metabolism as a pivotal battleground in the fight against malaria infection, especially given the emerging resistance to artemisinin [20,21,22], the primary treatment for malaria. Malaria parasites, as obligate intracellular organisms, heavily rely on reshaping host cells to ensure their survival. Notably, the occurrence of metabolic polymorphisms in malaria-endemic regions, such as G6PD deficiency [61], highlights how natural selection has historically provided solutions to combat malaria infection through adjustments in host metabolism [5,62].

Although heme degradation in nucleated cells is classically initiated by ER-anchored heme oxygenases (HMOX1 and HMOX2), mature human red blood cells represent a fundamentally different metabolic architecture. Terminally differentiated RBCs lack nuclei, endoplasmic reticulum, and mitochondria, precluding inducible or regulated de novo heme cleavage. Consistent with this, HMOX1/2 expression is minimal in the mature RBC proteome. However, mature RBCs retain high levels of downstream heme-handling components, including biliverdin reductases (BLVRA and BLVRB) and the iron exporter ferroportin (SLC40A1). The persistence of these proteins reflects a decoupled, downstream heme-handling system, rather than an intact canonical heme degradation pathway. Biliverdin reductases are abundant cytosolic enzymes with established antioxidant and redox-buffering functions [63] that operate independently of acute HMOX activity. These enzymes likely process biliverdin and heme-derived metabolites inherited from late erythropoiesis, generated through slow non-enzymatic heme oxidation during RBC aging, or delivered via circulating metabolites and microvesicles. Similarly, sustained ferroportin expression supports iron homeostasis during the prolonged circulatory lifespan of RBCs, preventing intracellular iron accumulation as hemoglobin undergoes gradual oxidative turnover. Thus, mature RBCs do not execute inducible heme degradation but instead maintain downstream detoxification and redox-management capacity, a modular metabolic configuration that becomes exploitable under pathological perturbations such as malaria infection.

RBCs are functionally robust cells, constituting the most abundant yet metabolically minimalist cell type in the human body [4,34]. Upon release from bone marrow development, RBCs lack nuclei and organelles for most cellular repair processes, yet circulate for approximately three months [34,35], equipped with a robust antioxidant system, including high levels of hemoproteins like catalase and cytochrome b5 reductase [33,34]. In the context of malaria infections, natural selection has favored mechanisms that increase redox stress to kill parasites while preserving normal RBC function, as evidenced by the worldwide high prevalence of hematological diseases like G6PD deficiency and sickle cell anemia [64,65,66]. Our aim is to exploit this redox stress using bait-and-kill approaches. Our unique approach selectively enhances host redox stress in infected cells. To further elucidate the mechanisms, we are investigating a range of human malaria-related hematological diseases to understand the role of inherent host metabolic polymorphisms in determining infection outcomes.

We acknowledge the work of Sigala et al. (2015) [14], who first demonstrated the potential of ALA-induced porphyrin accumulation for antimalarial therapy. Their research provided valuable insights into the mechanism of ALA uptake by infected red blood cells, which has informed our understanding of ALA-induced effects in our study. Although Sigala et al. focused on a photodynamic approach, their findings establish a foundational basis for our non-light-dependent “bait-and-kill” strategy.

We propose that the activation of artemisinin is primarily due to host-derived heme metabolites, which are present in orders of magnitude higher quantities than parasite-derived heme, as human erythroid cells contain approximately 90% of all human heme. Prior research, particularly the work of Sigala et al., supports the interpretation that heme metabolites in infected red blood cells are primarily of host origin. A limitation of our study is that the parasite also possesses an endogenous heme pathway and can contribute to the activation process. We clarify that both host and parasite heme are present in infected cells. Detailed biochemical studies are needed to fully elucidate the roles of host versus parasite heme in future research.

These findings challenge the assumption that ALA uptake is restricted to late-stage parasites and highlight the progressive nature of host membrane remodeling beginning shortly after invasion. ALA (molecular weight ≈ 131 Da) is a small, zwitterionic molecule, substantially smaller than DHA (≈284 Da), which is already active in early ring stages. This size advantage supports the plausibility of ALA entering infected RBCs prior to full New Permeability Pathway (NPP) maturation. Together with direct synergy observed in ring-stage parasites and extended culture showing no recrudescence, our results support a model in which early-stage ALA uptake contributes to the bait-and-kill mechanism.

Drawing inspiration from recent discoveries targeting similar pathways in cancer cells [9,10,11], we harness the resulting accumulation of redox-active heme intermediates [24,67] to effectively induce cell death. Moreover, there is potential for further exploration into strategies that exploit host redox stress during infection, offering novel ways in treating other types of intracellular pathogens.

This combinatorial approach could potentially extend to testing artemisinin in combination therapy with other frontline antimalarials [21], thereby revitalizing existing drugs that have encountered resistance. Testing the combination of ALA with other antimalarials, such as chloroquine, doxycycline, or clindamycin, could provide valuable insights. While our primary focus is on enhancing artemisinin’s efficacy due to its mechanism involving heme metabolites, exploring ALA in combination with other drugs may also contribute to new drug screening projects.

Given that malaria can infect diverse host cells, including liver [68,69,70] and bone marrow cells [71,72], which undergo parasite-induced remodeling, the bait-and-kill strategy holds promise for application across various stages of malaria infection. Evidence indicates that the heme biosynthesis pathway is dysregulated in infected liver and marrow niches, presenting opportunities to develop interventions that target multiple life stages of malaria beyond red blood cell infection. Further investigation could explore the efficacy of the bait-and-kill strategy other phases of malaria infection, thereby taking initial steps for therapeutic interventions against malaria by targeting different host metabolism.

## 4. Methods

### 4.1. Analysis of Cancer Gene Essentiality in Heme Biosynthesis Using CRISPR KO Data

The assessment of cancer gene essentiality via CRISPR/Cas9 was conducted by leveraging data available on the DepMap Portal, using established methodologies [73,74,75], similar to methods we previously used [9]. Whole-genome CRISPR/Cas9 datasets were utilized to identify notable reductions in the growth of mutant cells following targeted gene knockouts in pooled experiments. Gene essentiality was inferred based on the dependency of a particular gene, determined through CRISPR/Cas9 gRNA-mediated gene knockout. Essential scores were employed to evaluate cell growth fitness, with lower scores indicating a more significant impact on cell viability upon gene loss. Specifically, scores of 0, <0, and >0 represented no change in fitness, loss of fitness, and gain in fitness (suggesting potential growth advantage for the cell line) under the assay conditions. Identification of commonly essential genes was based on their significance for the fitness of most cell lines across various cancer types [76,77].

### 4.2. Analysis of Human Mature Red Blood Cell (RBC) Heme Biosynthesis and Malaria Parasite Host Remodeling Data

Hemopoiesis gene expression data were sourced from previously published datasets [25], focusing on various stages of erythropoiesis, such as common myeloid progenitor (MYE_0), megakaryocyte/erythroid progenitor (MYE_1 and MYE_2), and erythroid cells (ERY1-4). Quantitative proteomic data of human mature red blood cells (RBCs) were obtained from comprehensive datasets [1,2] comprising a total of 18,581 proteins present in RBCs. Within this dataset, approximately 1200 proteins constitute the canonical RBC proteome, including mid-steps of heme biosynthesis enzymes ALAD, HMBS, UROS and UROD. The full sets of RBC proteome exhibit a broad range of protein abundances, from high to low, with the lowest abundance represented by a single peptide, for example, the mitochondrial hemoprotein COX10. Proteins considered abundantly present were those ranking above the 0.75 percentile in peptide counts within RBC proteomes. Differential expression levels between early and late stages were assessed using the Wilcoxon test, with *p* values adjusted using the Benjamini–Hochberg method for multiple hypothesis testing.

For malaria host modeling protein analysis, the set of host-targeted proteins were retrieved from PlasmoDB v68 [78] by combining searches for the ‘PEXEL’ motif and ‘Host-Targeted’ motif in the reference *P. falciparum* 3D7 genome [15,16]. Gene essentiality data, represented by the Mutagenesis Index score, were obtained based on saturation transposon mutagenesis phenotypic data of parasite in vitro survival in blood stages.

Host permeability proteins were identified based on the Malaria Parasite Metabolic Pathway (MPMP) v2023 (http://mpmp.huji.ac.il, accessed in 13 December 2024), specifically focusing on the permeability of the membrane of infected RBCs. Malaria gene expression data were retrieved from transcriptomes of seven sexual and asexual life stages [44], including two gametocyte stages (II and V), ookinete, and four time points of erythrocytic stages (ring, early trophozoite, late trophozoite, and schizont). Bimodal distribution estimation was performed using histograms displaying two prominent peaks, and Kernel Density Estimation (KDE) was employed to estimate the probability density function of the data, providing a smoothed estimate of the underlying distribution and identifying multiple modes.

### 4.3. Primary Liver Cell and Hepatoma Cell Line Preparation

The human primary liver cells utilized in our study are purchased from commercial sources. These cells are obtained from donors under strict ethical guidelines and regulations established by the providers. Since these cells are anonymized and do not involve direct interaction with human subjects, ethics consent is not applicable in this scenario. In vitro culture of primary human hepatocytes began by sterilizing 384-well plates (Greiner, Monroe, NC, USA, Cat No. 781091) in a class II biosafety cabinet and placing them in a secondary container to prevent evaporation. The wells were coated with 40 μL of 15 μg μL^−1^ rat tail collagen I (Corning, Corning, NY, USA, Cat No. 354236) in sterile filtered 0.02 M acetic acid (Thermo Fisher Scientific, Waltham, MA, USA) and kept at 37 °C overnight. Before seeding, the wells were washed thrice with sterile phosphate-buffered saline (PBS) and filled with 20 μL of in vitro GRO^®^ CP plate medium (BioIVT, Woodbury, NY, USA Cat No. Z99029), supplemented with 1× Pen-Strep-Neo solution (100×, Fisher, Cat No. 15640-055, Waltham, MA, USA) and 20 μM gentamicin (1000×, Fisher, Waltham, MA, USA Cat No. 15-710-072). Cryopreserved primary human hepatocytes (BioIVT, Woodbury, NY, USA Cat No. M00995-P) were thawed by immersion in a 37 °C water bath for 2 min, sterilized with 70% ethanol in a sterile field, and added directly to 4 mL plate medium. Live and dead cells were quantified using trypan blue exclusion on a Neubauer improved hemocytometer. The hepatocyte density was adjusted to 1 × 10^3^ live cells μL^−1^, and 18 μL of the cell suspension was added to each well. Medium exchange with the GRO^®^ CP plating medium occurred thrice weekly.

Three hepatoma cell lines, namely HC-04, HepG2, and SNU449, were used as liver cancer cell lines for comparative analysis. These cryopreserved cell lines were thawed, suspended in a hepatocyte culture medium previously prepared, and transferred to T75 flasks coated with collagen (Corning, Corning, NY, USA, Cat No. 354236,) at a density of 5 μg/cm^2^. The culture medium consisted of a 1:1 (*v*/*v*) mixture of F12 base medium (Invitrogen, Carlsbad, CA, USA, Cat No. 11765-054) and MEM base medium (Invitrogen, CA, USA, Cat No. A10490-01), supplemented with 10% FBS (Hyclone, Logan, UT, USA, Cat No. SH30070), 1.0 M HEPES (Invitrogen, Carlsbad, CA, USA Cat No. 15630-080), and 200 mM glutamine (Invitrogen, Carlsbad, CA, USA, Cat No. 25030-081). The cells were cultured until reaching 70% confluence, with the medium being changed every other day. Once the desired confluence was attained, the cells were trypsinized using TrypLE™ Express Enzyme (1X) (Gibco, Grand Island, NY, USA, Cat No. 12605028), washed with hepatocyte culture medium, and then seeded at a density of 6000 cells/well in 384-well plates (Greiner, Monroe, NC, USA, Cat No. 781091), with each well receiving 20 μL of the aforementioned medium. Cells were then incubated either in the absence or presence of 1.0 mM ALA at 37 °C for 4 h under very low light conditions. During the last 45 min of incubation, a staining solution diluted in phenol-free, serum-free RPMI (Gibco, Grand Island, NY, USA, Cat No. 11835055), containing Hoechst 33,342 (Life Technologies, Carlsbad, CA, USA, Cat No. H3570) at a final concentration of 10 μM, was added to the cells.

### 4.4. Cellular PPIX Quantification

Quantification of intracellular protoporphyrin IX (PPIX) accumulation was performed using fluorescence-activated cell sorting (FACS), following established protocols [79]. Cells were cultured in medium as described in the Cell Culture section, supplemented with 1.0 mM 5-aminolevulinic acid (ALA), and maintained at 37 °C for 4 h under low light conditions. After incubation, cells underwent triple washing with Dulbecco’s phosphate-buffered saline (DPBS, 1X, Ca2+- and Mg2+-free; Corning, Corning, NY, USA, Cat No. 21-031-CV) and were resuspended in 250 μL of 1× DPBS. Subsequently, cells were washed once with serum-free medium (Gibco, Grand Island, NY, USA, Cat No. 11835055) and seeded in 6-well plates with the designated medium (as described in the Cell Culture section), with or without the addition of ALA, followed by incubation at 37 °C. Intracellular PPIX concentration was evaluated 18 h later using FACS. FACS analyses were conducted using a BD LSR II Analyzer (Becton, Dickinson, and Company) equipped with FACSDiva Version 6.1.3 software. To minimize background red fluorescence, the 633 nm-red laser was deactivated during PPIX emission data collection. PPIX emission within the 619 nm and 641 nm range (630/22BP filter) was measured following excitation with the 405 nm laser. Forward-scatter (FSC) versus side-scatter (SSC) dot plots were utilized to gate the entire cell population while excluding cell debris. A minimum of 10,000 cells within the gated region were then represented in dot plots of SSC vs. PPIX fluorescence, with the gate defined using cells lacking perturbation as negative controls.

### 4.5. Peripheral Blood Mononuclear Cell (PBMC) Isolation

Human peripheral blood mononuclear cells (PBMCs) were isolated from whole blood obtained from healthy donors using Ficoll-Paque density gradient centrifugation. Briefly, whole blood was diluted 1:1 with phosphate-buffered saline (PBS) and carefully layered over Ficoll-Paque solution (GE Healthcare, Chicago, IL, USA) in sterile conical tubes. Samples were centrifuged at 400× *g* for 30 min at room temperature with no brake. Following centrifugation, the PBMC layer at the plasma–Ficoll interface was carefully collected, transferred to a new tube, and washed twice with PBS to remove residual platelets and Ficoll. Cells were then resuspended in the appropriate culture medium and counted using trypan blue exclusion to assess viability before downstream assays.

### 4.6. Reactive Oxygen Species (ROS) Measurement by Flow Cytometry

Intracellular reactive oxygen species (ROS) levels were quantified using CellROX™ Orange Reagent (Thermo Fisher Scientific), a fluorogenic probe that exhibits increased fluorescence upon oxidation. Infected red blood cells (iRBCs) were cultured under standard conditions and treated with ALA (1 mM), dihydroartemisinin (DHA), or the ALA + DHA combination, as indicated for each experiment. Untreated infected cells were used as controls. Following drug treatment, cultures were incubated with CellROX Orange at the manufacturer-recommended concentration for 30 min at 37 °C, protected from light. Cells were then washed with phosphate-buffered saline (PBS) to remove excess dye and immediately analyzed by flow cytometry. Flow cytometric acquisition was performed on a BD LSR II flow cytometer using standard settings. CellROX Orange fluorescence was collected in the FITC channel. To identify infected red blood cells, SYBR Green was used to label parasite nucleic acids (excitation/emission 498/522 nm). Forward- and side-scatter parameters were used to gate intact cells and exclude debris. Data were analyzed using FlowJo software v10.6. All experiments were performed with multiple biological replicates (n > 3).

### 4.7. Malaria Parasite Culture

For the cultivation of asexual blood-stage parasites, O+ human red blood cells (RBCs) were utilized and maintained in a controlled environment at 37 °C with 5% CO_2_ humidity. The parasite culture medium consisted of RPMI 1640 supplemented with 25 mM NaHCO_3_, 11 mM glucose, 25 mM HEPES (pH 7.4), 0.367 mM hypoxanthine, and 5 μg/liter gentamicin. Additionally, 0.5% AlbuMAX II lipid-rich bovine serum albumin from Thermo Fisher Scientific, MA, was added to enhance the lipid content. To synchronize the parasites at the ring stage, a 5% D-sorbitol treatment method was employed.

As our study utilizes human blood purchased from blood banks and parasite strains devoid of any human host information, ethics consent is not applicable in this context. The human blood obtained from blood banks is anonymized and does not involve direct interaction with human subjects. Similarly, the parasite strains used in our research are devoid of any human host information and are maintained independently of human involvement. Therefore, no ethics consent is required for the use of these materials in our study.

### 4.8. Malaria Ring-Stage Survival Assays

The Ring-Stage Survival Assay (RSA) was conducted following established protocols. Initially, schizonts were purified from tightly synchronized cultures using a 75% Percoll gradient (Sigma-Aldrich, St. Louis, MO, USA). After purification, the schizonts were washed once in RPMI 1640 incomplete medium and allowed to rupture, invading fresh red blood cells (RBCs) for a duration of 3 h. Following invasion, the cultures underwent another synchronization process using sorbitol to select for early rings and eliminate any remaining schizonts. For the RSA_0–3_ h assay, ring-stage parasites (0 to 3 h post-invasion) at a parasitemia of 1% and hematocrit of 1% were exposed to 700 nM Dihydroartemisinin (DHA) for a period of 6 h, followed by a single wash.

Subsequently, the cultures were allowed to incubate for 66 h, after which approximately 10,000 RBCs were blindly counted on thin blood smears to determine the number of viable parasites. For ALA combination assays, 1 mM ALA was added along with 700 nM DHA to the 0–3 h rings and aliquoted in four distinct wells. 1 mM ALA was either replenished after DHA washout on day 1 or day 1 & 2 or all 3 days until readout.

To evaluate whether ALA alone has any antimalarial activity or cytotoxic effect under the same conditions as the combination treatment, synchronized ring-stage parasites were exposed to 1 mM ALA. ALA was added either once at the beginning of the assay or replenished daily for consecutive days to mimic cumulative exposure in the combination experiments. Parasite cultures were maintained under standard conditions for >48 h before parasitemia was assessed by Giemsa-stained thin smears and flow cytometry.

Smears were prepared at 72 h, and further parasite growth was monitored through Geimsa stained smears for over 3 weeks. For all assays, parallel dimethyl sulfoxide (DMSO)-treated controls (0.1% concentration) were included, and survival rates were expressed as ratios of viable parasites in DHA-exposed or DHA + ALA exposed vs. DMSO-exposed samples. All assays were performed in 3 biological replicates.

## 5. Statistical Analysis

All experiments were performed with biological replicates. Unless otherwise stated, experiments were conducted with at least three independent biological replicates (n ≥ 3). Quantitative data are presented as mean ± standard error of the mean (SEM).

For comparisons between two groups, statistical significance was assessed using two-tailed nonparametric tests (Mann–Whitney U test). For comparisons involving multiple experimental conditions, one-way analysis of variance (ANOVA) followed by appropriate post hoc testing was used. No data points were excluded from the analysis unless technically invalid (e.g., acquisition failure).

## Data Availability

The original contributions presented in this study are included in the article/Supplementary Material. Further inquiries can be directed to the corresponding author(s).

## Supplementary Materials

The following supporting information can be downloaded at: https://www.mdpi.com/article/10.3390/ph19010167/s1, Figure S1: Testing ALA and Artemisinin Synergy in Human Cell Line; Figure S2: Flow cytometric analysis of ALA- and dihydroartemisinin (DHA)-induced PPIX accumulation and ROS production. Table S1: Gene expression levels of terminal erythropoiesis related genes. (Eight erythropoiesis developmental stages were analyzed for heme related metabolism in erythroid); Table S2: Human mature red blood cell proteome analysis related to heme metabolism; Table S3: *P. falciparum* host remodel gene analysis. (Data retrieved from PlasmoDB v68); Table S4: Drug synergy experimental raw results of DHA and ALA interaction in killing HC-04 liver cancer cell line. Experimental triplicates were performed as listed in 3 plates data; Table S5: ALA dosage for safety and efficacy in vitro, in vivo and in human clinical studies. Refs [80,81] are listed here.
