# Supplementary material for: Targeting Infected Host Cell Heme Metabolism to Kill Malaria Parasites
Source: Pharmaceuticals (Basel). 2026 Jan 17;19(1):167. doi: 10.3390/ph19010167 (PMC12845165; doi:10.3390/ph19010167)
Supplement: Supplementary file 1 [file pharmaceuticals-19-00167-s001.zip › Supplemental Figure S1.pdf]

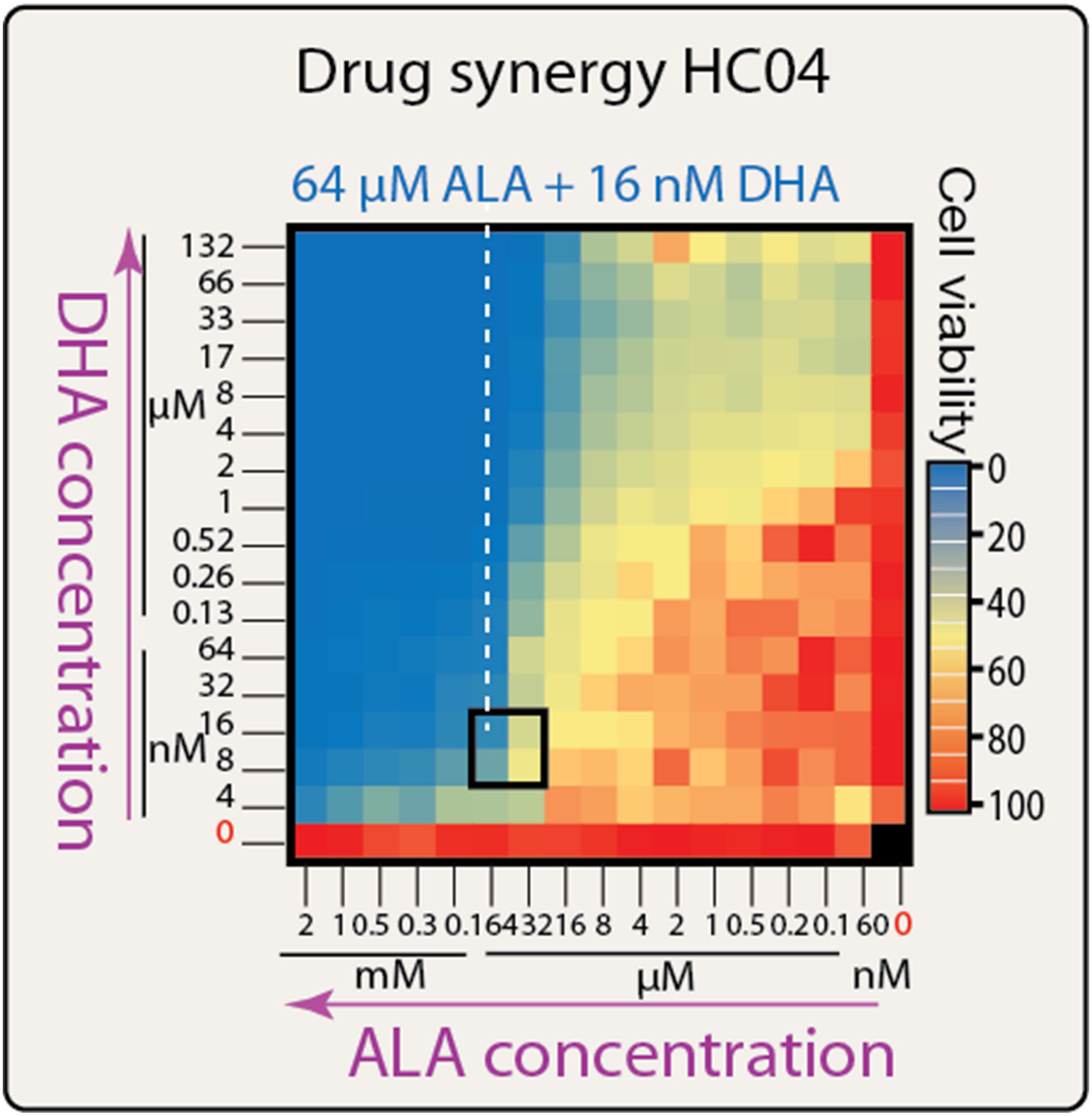
Drug synergy HC04
64 µM ALA + 16 nM DHA
Cell viability
0
20
40
60
80
100
132
66
33
17
8
4
2
1
0.52
0.26
0.13
64
32
16
8
4
0
µM
nM
DHA concentration
2 1 0.5 0.3 0.16 64 32 16 8 4 2 1 0.5 0.2 0.1 60 0
mM
µM
nM
ALA concentration

Supplemental Figure S1

Testing ALA and Artemisinin Synergy in Human Cell Line

Drug synergy between ALA and DHA in human liver HC-04 cells demonstrates potent synergistic effects in killing cancer cells. A range of drug concentrations were assessed, from nanomolar (nM) to 133 micromolar (μM) for DHA, and from nanomolar (nM) to millimolar (mM) for ALA. This drug-drug interaction study was conducted in experimental triplicates. While neither ALA nor DHA alone showed toxicity at the highest tested doses against HC-04 cells, their combination effectively kills cancer cells, even at low concentrations of DHA in the nanomolar range. (DHA: dihydroartemisinin, the active metabolite of artemisinin)
