# Supplementary material for: Targeting Infected Host Cell Heme Metabolism to Kill Malaria Parasites
Source: Pharmaceuticals (Basel). 2026 Jan 17;19(1):167. doi: 10.3390/ph19010167 (PMC12845165; doi:10.3390/ph19010167)
Supplement: Supplementary file 1 [file pharmaceuticals-19-00167-s001.zip › Supplemental Figure S2.pdf]

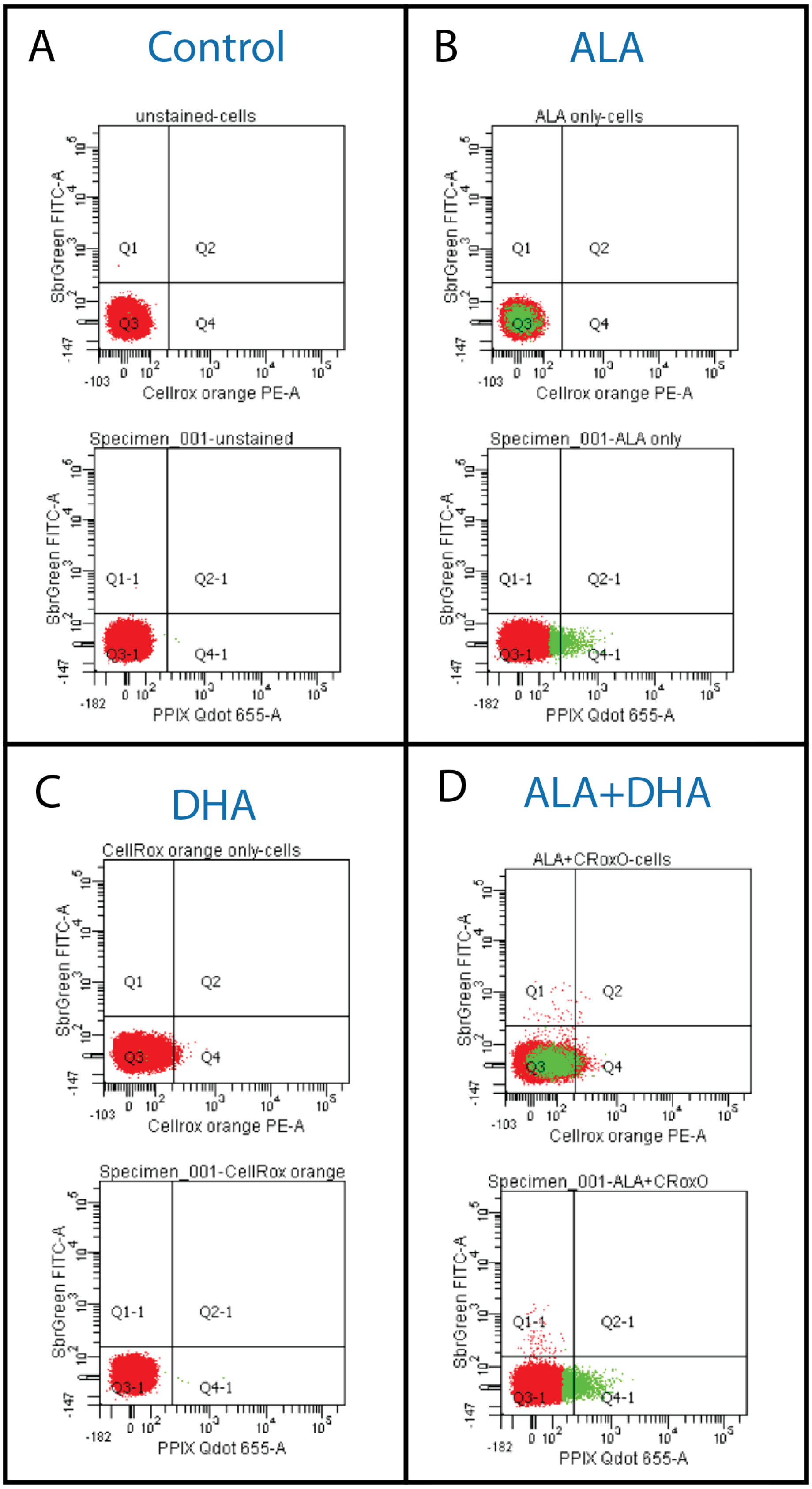
A
Control
unstained-cells
SbrGreen FITC-A
Cellrox orange PE-A
Specimen_001-unstained
PPIX Qdot 655-A
B
ALA
ALA only-cells
Specimen_001-ALA only
C
DHA
CellRox orange only-cells
Specimen_001-CellRox orange
D
ALA+DHA
ALA+CRoxO-cells
Specimen_001-ALA+CRoxO
Q1
Q2
Q3
Q4
Q1-1
Q2-1
Q3-1
Q4-1

Supplemental Figure S2. Flow cytometric analysis of ALA- and dihydroartemisinin (DHA)-induced PPIX accumulation and ROS production

Flow cytometry was used to assess protoporphyrin IX (PPIX) accumulation and reactive oxygen species (ROS) production in *P. falciparum* 3D7–infected red blood cells under the indicated treatment conditions.
(A) Untreated control (no ALA, no DHA). Baseline levels of both PPIX and ROS are observed.
(B) ALA treatment (1 mM) alone induces a distinct population with elevated PPIX fluorescence but does not increase ROS levels.
(C) DHA treatment (700 nM) alone increases intracellular ROS levels without inducing detectable PPIX accumulation.
(D) Combined ALA (1 mM) and DHA treatment results in simultaneous increases in both PPIX accumulation and ROS production.
For all panels, the Y-axis indicates FITC channel. The X-axis represents PPIX fluorescence (lower panels) or CellROX Orange (upper panels), as indicated. PPIX fluorescence was detected using excitation at 405 nm and emission collected in the red channel (633 nm). ROS was measured using CellROX Orange. SYBR Green was used to label parasite nucleic acids (excitation/emission 498/522 nm) to identify infected red blood cells. Dihydroartemisinin (DHA), the active metabolite of artemisinin, was used at the indicated concentration.
