# Supplementary material for: Targeting Infected Host Cell Heme Metabolism to Kill Malaria Parasites
Source: Pharmaceuticals (Basel). 2026 Jan 17;19(1):167. doi: 10.3390/ph19010167 (PMC12845165; doi:10.3390/ph19010167)
Supplement: Supplementary file 1 [file pharmaceuticals-19-00167-s001.zip › Supplemental Table S5.pdf]

Supplemental Table S5

ALA dosage for safety and efficacy in vitro, in vivo and in human clinical studies

Experimental system	ALA dosage	Disease model	ALA effect	References
Mouse model	300 mg/kg	Imaging	No toxicity. Induced porphyrin synthesis and accumulation in cancers.	Unpublished data
Mouse model	40-100 mg/kg	Imaging	No toxicity. Induced porphyrin synthesis and accumulation in cancers.	Predina et al [1]
Mouse model	80 mg/kg	Brain cancer treatment	No toxicity. Treatment of brain tumor in combination with DHA.	Stowers et al [2]
Mouse model	300-600 mg/kg	Malaria treatment	No toxicity. Improved treatment of malaria	Suzuki et al [3]
Human organoid	65.5 μM ALA	Brain cancer treatment	No toxicity. Synergistic cancer killing with DHA	Stowers et al [2]
Human	20 mg/kg (current FDA recommended dose)	Brain cancer surgery imaging	No toxicity. Induced porphyrin synthesis and accumulation for cancer imaging, PPIX lasting at least 2-4 hours, over 58,000 patients globally have received ALA since its approval in Europe in 2007	FDA Gleolan report NDA 208630
Human	up to 2250 mg/day for 3-7 days	COVID treatment in Japan	No toxicity. Improved COVID patient recovery	Kaketani and Nakajima [4]
Human	200 mg/day for 3 months	Type 2 diabetes treatment	No toxicity. No diabetes disease improvement found.	Al-Saber et al [5]

Human	40 mg/kg	ALA pharmacodynamics study	No toxicity. Induced porphyrin synthesis and accumulation in patient (precancerous skin lesions) serum, peaking at 742 µg/l after ~7 hours oral ALA, 95% PPIX elimination after 35 hours.	Rick et al [6]
Human	60 mg/kg	ALA pharmacodynamics study	No toxicity. Induced porphyrin synthesis and accumulation in patient (GI track cancers) serum peaking at 1500 -3300 µg/l after ~8 hours oral ALA, complete PPIX elimination after 48 hours.	Webber et al [7]
Human (control group)	0	Healthy human controls	Healthy human plasma background PPIX levels 0-8 µg/l	Rick et al [6]

Abbreviations: ALA, 5-aminolevulinate; DHA, dihydroartemisinin; PPIX, protoporphyrin IX

References:

1. Predina JD, Runge J, Newton A, Mison M, Xia L, Corbett C, Shin M, Sulyok LF, Durham A, Nie S *et al*: **Evaluation of Aminolevulinic Acid-Derived Tumor Fluorescence Yields Disparate Results in Murine and Spontaneous Large Animal Models of Lung Cancer**. *Scientific Reports* 2019, **9**(1):7629.
2. Taubenschmid-Stowers J, Orthofer M, Laemmerer A, Krauditsch C, Rózsová M, Studer C, Lötsch D, Gojo J, Gabler L, Dyczynski M *et al*: **A whole-genome scan for Artemisinin cytotoxicity reveals a novel therapy for human brain tumors**. *EMBO Mol Med* 2023, **15**(3):e16959.
3. Suzuki S, Hikosaka K, Balogun EO, Komatsuya K, Niikura M, Kobayashi F, Takahashi K, Tanaka T, Nakajima M, Kita K: **In vivo curative and protective potential of orally administered 5-aminolevulinic acid plus ferrous ion against malaria**. *Antimicrob Agents Chemother* 2015, **59**(11):6960-6967.
4. Kaketani K, Nakajima M: **Case Reports: Safety, Tolerability, and Efficacy of 5-Aminolevulinic Acid Phosphate, an Inducer of Heme Oxygenase 1, in Combination with Sodium Ferrous Citrate for the Treatment of COVID-19 Patients**. *The Open COVID Journal* 2021.
5. Al-Saber F, Aldosari W, Alselaiti M, Khalfan H, Kaladari A, Khan G, Harb G, Rehani R, Kudo S, Koda A *et al*: **The Safety and Tolerability of 5-**

Aminolevulinic Acid Phosphate with Sodium Ferrous Citrate in Patients with Type 2 Diabetes Mellitus in Bahrain. *J Diabetes Res* 2016, **2016**:8294805.
6. Rick K, Sroka R, Stepp H, Kriegmair M, Huber RM, Jacob K, Baumgartner R: **Pharmacokinetics of 5-aminolevulinic acid-induced protoporphyrin IX in skin and blood**. *J Photochem Photobiol B* 1997, **40**(3):313-319.
7. Webber J, Kessel D, Fromm D: **Plasma levels of protoporphyrin IX in humans after oral administration of 5-aminolevulinic acid**. *J Photochem Photobiol B* 1997, **37**(1-2):151-153.
